# C-reactive protein, Neopterin and Beta_2_ microglobulin levels pre and post TB treatment in The Gambia

**DOI:** 10.1186/s12879-016-1447-9

**Published:** 2016-03-08

**Authors:** Joseph Mendy, Toyin Togun, Olumuyiwa Owolabi, Simon Donkor, Martin O. C. Ota, Jayne S. Sutherland

**Affiliations:** Vaccines & Immunity Theme, Medical Research Council (MRC) Unit, Atlantic Road, Fajara, The Gambia; Current address: World Health Organisation Regional Office, Brazzaville, Congo; MRC Unit, PO Box 273, Banjul, The Gambia

**Keywords:** Activation markers, TB therapy, Point-of-care tests

## Abstract

**Background:**

Tuberculosis is one of the leading causes of morbidity and mortality in developing countries. Analysis of the host immune response may help with generating point-of-care tests for personalised monitoring. Thus, the aim of this study was to assess the relationship between immune activation markers: C-reactive protein (CRP), Beta_2_ microglobulin (B_2_M) and Neopterin, disease severity prior to treatment and response to therapy in adult pulmonary TB patients.

**Methods:**

HIV negative adult pulmonary TB index cases (*n* = 91) were recruited from the TB clinic at MRC, The Gambia. Plasma samples were collected at enrolment and at 2 and 6 months following TB treatment initiation. An enzyme linked immunosorbent assay (ELISA) was performed for evaluation of CRP, B_2_M and Neopterin levels and correlated with clinical and microbiological parameters including strain of infection. Disease severity was determined using Chest X-ray (CXR), Body Mass Index (BMI) and sputum smear grade.

**Results:**

Plasma levels of all three markers were highly elevated in patients at recruitment and declined significantly during TB therapy. No correlation with disease severity was seen at recruitment. CRP showed the most significant decrease by 2 months of treatment (*p* < 0.0001) whereas levels of B_2_M and Neopterin showed little change by 2 months but a significant decrease by 6 months of treatment (*p* = 0.0002 and *p* < 0.0001 respectively). At recruitment, B_2_M levels were significantly higher in subjects infected with *Mycobacterium africanum (Maf)* compared with those infected with *Mycobacterium tuberculosis* sensu stricto *(Mtb)* (*p* = 0.0075). In addition, while CRP and Neopterin showed a highly significant decline post-treatment regardless of strain (*p* < 0.0001 for all), B_2_M showed differential decline depending on strain (*p* = 0.0153 for *Mtb* and *p* = 0.0048 for *Maf*) and levels were still significantly higher at 6 months in *Maf* compared to *Mtb* infected subjects (*p* = 0.0051).

**Conclusion:**

Our findings suggest that activation markers, particularly CRP, may have a role in identifying good response to TB therapy regardless of the strain of infection and could be further developed as point-of-care tests. In addition, B_2_M levels may allow differentiation between *Mtb* and *Maf*-infected subjects.

## Background

Tuberculosis (TB) is one of the leading global causes of morbidity and mortality, particularly in Sub-Saharan Africa with 1.5 million deaths per year [1]. Despite many advances in treatment and campaigns such as Directly Observed Treatment Short course (DOTS) by the World Health Organisation (WHO), difficulty in diagnosis, non-adherence to treatment, emergence of multi-drug resistance (MDR) strains and TB/HIV co-infection have all fuelled the spread of the disease [2]. The causative pathogen, *Mycobacterium tuberculosis complex (MTBC)* includes *M. tuberculosis (Mtb), M. africanum (Maf), M. canettii, M. bovis, M. microti, M. caprae, M. orygis, and M. pinnipedii* [3] with *Mtb* and *Maf* the most common strains in human diseases. In West Africa about half of pulmonary TB cases are caused by *Maf* [4–6]. Though the disease produced by both strains looks similar [4], they have significant differences in their genomes particularly in the Region of Difference 1 (RD1) containing crucial T cell epitopes. In this regard, *Maf* infected subjects were observed to be less responsive to both ESAT-6 and TST compared to *Mtb* infected counterparts [5] and are more likely to have severe cavitation on chest x-ray [5]. Two recent studies from Gambia have shown comparable immune profiles regardless of infecting strain at baseline but significant divergence post-treatment at the cellular [7], metabolomic [8] and transcriptomic [8] levels. Due to the intensive nature of anti-TB therapy and these differences in therapy response depending on the strain of infection [7, 8], a point-of-care test that allows personalised monitoring of treatment response would be beneficial.

Previous studies analysing response to therapy have focussed on either C-reactive protein (CRP) or cytokine/chemokine profiles. A recent meta-analysis of cytokine markers has shown marked variation between studies due to low subject numbers and differences in follow-up times [9]. Activation markers such as CRP, Neopterin and Beta_2_ microglobulin (B_2_M) may hold the key for a fast, effective method of treatment monitoring at the point-of-care setting. CRP is an acute phase reactant and is a robust indicator of immune system activity. Its serum concentration has been used as a marker of inflammation in patients with diabetes mellitus [10], tuberculosis [11] and asthma [12]. Breen et al found that an elevated CRP detected 85 % of proven tuberculosis cases in the UK [13] raising the possibility of using CRP as a point-of care test. Neopterin is released by monocytes/macrophages when activated by Interferon-γ (IFN-γ) and acts as a mediator of cell immunity against intracellular pathogens [14]. Previous studies have shown increased serum Neopterin level in HIV/TB co-infected patients before anti-TB treatment and a decline after treatment [15]. In another study, serum Neopterin and IL-2 were observed to decline with TB treatment [16, 17]. B_2_M is a component of major histocompatibility (MHC) class 1 molecules found on the surface of all nucleated cells. It is also found in a free state in various body fluids suggesting an influence in disease pathology with a rise observed in the presence of glomerular impairment, or lymphocyte activation [16]. Past studies have shown a correlation with HIV disease progression [18, 19] but few studies have evaluated its role in TB. One study did, however, show clinical utility for treatment monitoring in HIV negative TB patients, particularly those with high levels of B_2_M at presentation [20].

Identification of markers that correlate well with response to TB therapy, will aid in the development of tests to improve personalised medicine. Thus the aim of this study was to determine the levels of CRP, Neopterin and B_2_M in relation to pulmonary tuberculosis disease severity at recruitment and changes in response to TB treatment in a cohort of TB patients from The Gambia. Due to the prevalence of *Mycobacterium africanum (Maf)* in this region [8], we were also able to determine differential responses based on the strain of infection before and after treatment initiation.

## Methods

### Study participants

Ninety-one adult pulmonary TB index cases with newly diagnosed smear-positive pulmonary TB were recruited from the TB clinic at the Medical Research Council (MRC) Unit, The Gambia. Participants provided written informed consent prior to collection of samples. Clinical evaluation, symptom screening, Body Mass Index (BMI), and chest posterior-anterior x-ray were performed and heparinised blood and sputum were collected for immunological and microbiological evaluations respectively. A Chest X-ray showing 1–2 infected lobes was considered minimal; 3 to 4 infected lobes with 1 cavitation considered moderate; and 5–6 lobes infected with cavitation considered advanced disease (for analysis purposes a nominal scale of 1–6 was used to define the number of lobes infiltrated). All subjects were treated according to the Gambian National Tuberculosis and Leprosy Control Programme’s conventional therapy of 2 months intensive treatment with Isoniazid, Rifampicin, Pyrazinamide, Ethambutol, followed by a second phase of four months with only Isoniazid and Rifampicin (2HRZE/4HR). Following initiation of standard TB treatment, participants were followed up at 2 and 6 months for further blood draw and sputum smear microscopy and culture to determine treatment response. Chest x-ray was performed at 2 months but not at 6 months follow-up. All specimens sent for mycobacterial confirmation were stained for acid-fast bacilli (AFB) by Ziehl-Neelsen (ZN) stain and cultured in liquid culture media (BACTEC MGIT, Becton Dickinson, USA). Spoligotyping was also performed to determine the strain of infection as previously described [8]. By the end of treatment all study subjects were confirmed microbiologically cured (negative) by sputum culture. The study was approved by the Gambian government/MRC joint ethics committee.

### Sample preparation

Plasma was collected and stored at -20 °C prior to analysis. On the day of sample analysis, the frozen plasma was thawed and centrifuged at 1500 rpm to remove debris.

### Evaluation of activation markers in plasma

Samples were evaluated using commercially available kits for plasma CRP, B_2_M (both from Immunology Consultants Laboratory, USA), and Neopterin (IBL International, Germany). ELISAs were performed according to the manufacturer’s instructions. All samples were run in duplicates and analysed using Softmax Pro 4.7.1 (Molecular Devices, USA). A four-parameter logistic curve was used to interpolate test samples values from the standard curve at 450 nm wave length.

### Measurement of C-reactive protein and Beta_2_ microglobulin

The microtiter plates provided were pre-coated with monoclonal antibodies specific to CRP or B_2_M. Samples were added to each well in duplicate and incubated for 15 min. A washing step was performed to remove the unbound proteins then secondary antibodies conjugated with horseradish peroxidise (HRP) were added for a further 15 mins. Following another wash, chromogenic substrate 3, 3’, 5’, 5’-tetramethylbenzidine (TBM) was added and incubated for 10 min, resulting in a colour change. A colour development stop solution (0.3 M sulfuric acid) was then added and the plate read at 450 nm.

### Measurement of neopterin

The Neopterin concentrations were determined using a competitive ELISA. Microtiter strips were pre-coated with goat-anti-rabbit Neopterin antibody. 20 μl each of sample, standard or controls were added to appropriate wells and incubated for 90 mins. 100 μl of enzyme conjugate and 50 μl of neopterin antiserum were then added to each well. Following a wash to remove unbound antibodies, 150 μl of TMB substrate solution was added and incubated at room temperature (RT) for 10 mins. After the incubation, the reaction was stopped by the addition 150 μl of TBM Stop Solution and briefly mixed by gently shaking the plate. The concentration of Neopterin was measured within 15 min at 450 nm optical density. The results were acceptable only when the controls were within defined ranges.

### Statistical analysis

The results were analysed with GraphPad Prism 6 (Software MacKiev, USA) using Wilcoxon Ranked sum test for paired values (ie pre and post-treatment for the same subject) or Mann-Whitney U-test for between subject comparisons (ie *Maf* versus *Mtb*-infected). Multivariable analysis was performed to adjust for age, sex and BMI using SPSS (v22, IBM, USA). A p-value of ≤0.05 was considered significant. Correlation with clinical and microbiological parameters was performed using Spearman’s correlation test.

## Results

### Subject information

Samples from 91 HIV negative subjects analysed (Table 1). The median (interquartile range (IQR)) age was 29 years (22–38) and 64 % were male. Body Mass Index (BMI) at recruitment was (median (IQR)) 18.6 kg/m^2^ (17.3–19.3) and this significantly increased by 6 months of treatment (19.6 kg/m^2^ (1.6–22.1; *p* = 0.0374; Table 1). The majority of subjects at recruitment had a smear grade of 3 (50 %), with all samples negative by 2 months for those analysed (not all patients could produce sputum for analysis at 2 and 6 months). CXR scores were highly variable with the majority of subjects having moderate to extensive disease at recruitment (79 %), which resolved to 45 % having minimal and 55 % moderate disease by 2 months, and 83 and 17 % with minimal versus moderate disease by 6 months (for those with readings available; Table 1).

**Table 1:** Patient characteristics at recruitment, 2 and 6 months of treatment

Time-point	Recruitment	2 months	6 months
N=	91	91	91
Age (median (IQR))	29 (22–38)	-	-
Males n(%)	58(64)	-	-
BMI (median (IQR))	18.6 (17.3–19.3)	19.1 (17.2–21.8)	19.6 (18.6–22.1)*
Smear grade n (%)			
Undetectable	0(0)	57(62)	22(24)
1+	24(26)	0(0)	0(0)
2+	22(24)	0(0)	0(0)
3+	46(50)	0(0)	0(0)
Unknown	0(0)	35(38)	69(76)
X-ray score n (%)			
Minimal	19(21)	30(33)	24(26)
Moderate	50(55)	36(40)	5(5)
Extensive	22(24)	0(0)	0(0)
Unknown	0(0)	24(26)	63(69)

* = *p* < 0.05 compared to pre-treatment; *BMI* body mass index, *IQR* interquartile range

### CRP, Neopterin and B_2_M levels pre and post-treatment

**Fig. 1:**
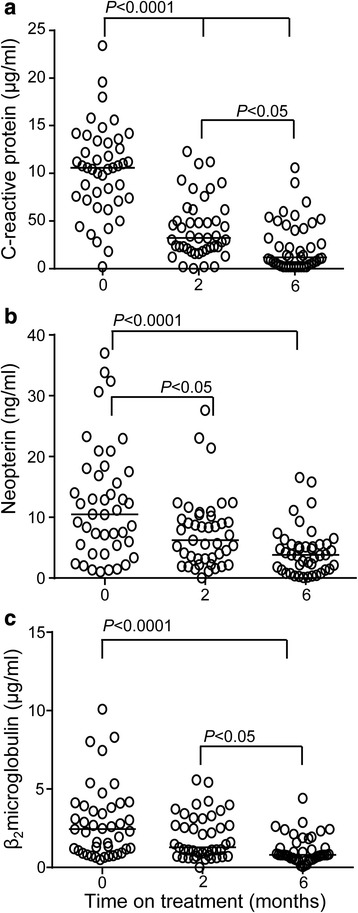
Decrease in plasma activation marker levels in response to TB therapy. Plasma from adult TB cases was assessed by ELISA for levels of C-reactive protein (**a**), Neopterin (**b**) and Beta_2_-microglobulin (**c**) before and after initiation of TB therapy. *n* = 91. Bar indicates median. Data were analysed using Friedman repeated measures test with post-test comparisons

At recruitment, CRP had significantly higher levels than the other two markers (median (IQR) = 108(64–158) μg/ml compared to 13(8–24) ng/ml for Neopterin and 2.9(1.9–4.1) μg/ml for B_2_M (Fig. 1a–c). We found no correlation with CXR, BMI or smear grade for any of the activation markers at recruitment (data not shown). Plasma levels of all three markers were significantly reduced during anti-tuberculosis therapy. CRP showed the most notable decrease by 2 months of treatment; (*p* < 0.0001; Fig. 1a) and this was further reduced by 6 months (*p* < 0.0001 compared to baseline and *p* = 0.0029 compared to 2 months; Fig. 1a). In contrast, B_2_M showed a gradual decline by 2 months (*p* = 0.0234) with a more significant decline by 6 months (*p* = 0.0002 compared to baseline and *p* = 0.0036 compared to 2 months; Fig. 1c). Neopterin showed no significant difference by 2 months but was significantly decreased by 6 months compared to both baseline (*p* < 0.0001) and 2 months (*p* = 0.0218; Fig. 1b). By 6 months, the mean (SD) fold change from baseline was 35.6(55.0) for CRP, 3.9 (4.9) for Neopterin and 1.4 (0.5) for B_2_M. These values were still highly significant after adjusting for age, sex and BMI (data not shown).

### Differential expression of activation markers in subjects with different strains of infection

**Fig. 2:**
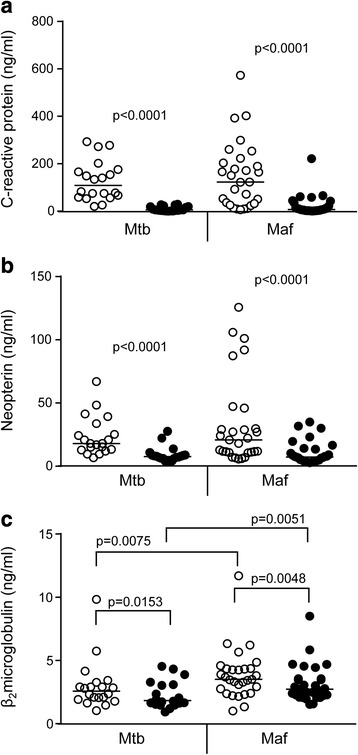
Differential levels of activation markers depending on the strain of infection. Plasma from adult TB cases was assessed by ELISA for levels of C-reactive protein (**a**), Neopterin (**b**) and Beta_2_-microglobulin (**c**) before and after initiation of TB therapy. The strain of infection was determined using spoligotyping. Maf = *Mycobacterium africanum*; Mtb = *Mycobacterium tuberculosis* sensu stricto. *n* = 29 for both groups. Bar indicates median. Data were analysed using Wilcoxon ranked sums test within groups and Mann-Whitney U-test between groups

We next analysed subjects based on their strain of infection. Both CRP and Neopterin showed no difference in levels between *Mtb* and *Maf* infected subjects (Fig. 2a and b) with both showing a highly significant decrease between baseline and 6 months. However, analysis of B_2_M levels showed significant differences in *Mtb* versus *Maf*-infected subjects at both recruitment and 6 month time-points. Levels in *Maf*-infected subjects were higher at both time-points compared to *Mtb* (median[IQR] = 2.7[1.96–3.17] and 1.8[1.5–3.1] for *Mtb* at recruitment and 6 months compared to 3.5[2.6–4.4] and 2.7[2.2–3.6] for *Maf* at recruitment and 6 months (*p* = 0.0075 and *p* = 0.0051 for *Mtb* versus *Maf* at 0 and 6 months respectively; Fig. 2c). We also found a significant but differential decline at 6 months depending on the strain of infection (*p* = 0.0153 and *p* = 0.0048 for *Mtb* and *Maf*-infected subjects respectively).

## Discussion

Analysis of plasma activation markers before and after initiation of anti-TB treatment has not been studied previously in The Gambia. We analysed HIV negative adults with confirmed pulmonary TB at recruitment, at 2 months (completion of the intensive phase of chemotherapy) and at 6 months (completion of the continuation phase of treatment). By 6 months, all subjects’ sputum samples were culture negative for AFB, indicating treatment success; and this correlated with significantly lower levels of plasma CRP, Neopterin and B_2_M. CRP showed the sharpest decline by 2 months of treatment whereas Neopterin and B_2_M had a gradual decline within the first 2 months, then a sharper decline between 2 and 6 months. When subjects were analysed based on their strain of infection no difference was seen in CRP and Neopterin levels but B_2_M levels were significantly higher in *Maf*-infected compared to *Mtb*-infected subjects at both recruitment and 6 months of therapy. Our data indicate that CRP may be a useful marker for pulmonary TB treatment monitoring and future studies should analyse earlier time-points post-treatment initiation. In addition, B_2_M is the first immune activation marker shown to differentiate between *Maf* and *Mtb*-infected subjects at recruitment and follow-up. These differences may relate to the reduced ability of *Maf* to secrete ESAT-6 [5], which has been shown to affect B_2_M production [21]

One of the major predictors of response to therapy in TB is disease severity prior to treatment initiation [22]. In our cohort, there was no correlation between any of the activation markers studied and extent of disease by CXR or smear grade at recruitment. We also did not see a relationship between levels of any of the markers at treatment and subsequent decline post-treatment. It is possible that measurement of the markers at shorter intervals and earlier time points might detect differences in the decline according to the initial values.

Our study supports previous findings showing high levels of CRP in patients at enrolment and a decline with treatment. One subject showed an increase in their CRP levels but a decline in Neopterin and B_2_M following treatment initiation. The reason for this is unclear but the subject may have had another concurrent pro-inflammatory condition that only elicited changes in CRP. A study with a larger sample size that includes drug resistant cases and measurements at shorter intervals may help to fully evaluate the clinical utility of each of the markers. Interestingly, there was no difference in levels of CRP and Neopterin in subjects infected with different strains of bacteria. West Africa has a prevalence of *Maf* infection [8], which has been shown to be less virulent than *Mtb* resulting in different clinical presentations and response to therapy. Interestingly, no studies to date have been able to find differences in any immunological or transcriptomic marker at recruitment between these two strains [7, 8]. Our finding that B_2_M can differentiate between *Maf* and *Mtb* infected subjects both at recruitment and after therapy opens new avenues for monitoring of immunity based on different strains. Further work should include subjects with other strains of infection such as Beijing, which is highly virulent [23].

## Conclusions

In conclusion, we have shown the clinical utility of three activation markers for monitoring of pulmonary TB treatment responses in The Gambia. Our findings suggest that activation markers, particularly CRP, may have a role in identifying good response to TB therapy regardless of the strain of infection and could be further developed as point-of-care tests. In addition, we have been able to describe for the first time a marker, B_2_M which can differentiate between *Mtb* and *Maf*-infected subjects.

## Abbreviations

CRP: C-reactive protein
B_2_M: BETA-2-Microglobulin
*Mtb*: *Mycobacterium tuberculosis sensu stricto*
*Maf*: *Mycobacterium africanum*
ELISA: enzyme-linked immunosorbent assay
TB: tuberculosis
HIV: human immunodeficiency virus
MRC: Medical Research Council
IQR: interquartile range
BMI: body mass index
